# Invasive mucormycosis during treatment for acute lymphoblastic leukaemia—successful management of two life-threatening diseases

**DOI:** 10.1007/s00520-019-04962-3

**Published:** 2019-08-14

**Authors:** Andreas Trobisch, R. Marterer, G. Gorkiewicz, S. Flaschberger, H. Lackner, M. Seidel, D. Sperl, A. Karastaneva, B. Kohlmaier, M. Egger, C. Urban, M. Benesch, V. Strenger

**Affiliations:** 1 Department of Paediatrics and Adolescent Medicine, Division of Paediatric Haematology/Oncology, Medical University, Graz, Austria; 2 Department of Paediatrics and Adolescent Medicine, Division of Neonatology, Medical University, Graz, Austria; 3 Department of Radiology, Division of Paediatric Radiology, Medical University Graz, Graz, Austria; 4 Diagnostic & Research Institute of Pathology, Medical University, Graz, Austria; 5 Department of Paediatrics, Klinikum Klagenfurt am Wörthersee, Klagenfurt, Austria; 6 Department of Paediatrics and Adolescent Medicine, Division of Paediatric Pulmonology and Allergology, Medical University, Graz, Austria; 7 Research Unit Infectious Diseases in the Immunocompromised Host, Medical University, Graz, Austria

**Keywords:** ALL, Mucormycosis, IFD, Antimycotic treatment, Chemotherapy

## Abstract

A 5-year-old patient treated for acute lymphoblastic leukaemia (ALL) developed proven pulmonary invasive fungal disease (IFD) due to *Actinomucor elegans*. While completing ALL treatment according to AIEOP ALL protocol 2009 for further 15 months, antifungal treatment with liposomal amphotericin B and intermittent additional posaconazole was continued until immune reconstitution 7 months after the end of ALL treatment. Repeated imaging guided treatment decisions. Twenty-six and 19 months after the end of ALL treatment and antifungal treatment, respectively, the patient is still in the first complete remission and shows no signs of active invasive fungal disease (IFD).

## Introduction

Invasive mould diseases remain an important cause of death in immunocompromised patients [1, 2]. While invasive diseases with *Aspergillus* sp. have mortality rates of 20–50%, mortality rates in mucormycosis range from 50 to 100% [3–5]. Differentiation between mucormycoses and other mould infections remains challenging, since these pathogens are difficult to culture and show similar radiological and clinical features leading from 4% to up to 90% of suspected mucormycosis cases not being confirmed until post-mortem examination [1, 4, 5]. Moreover, mucormycetes lack susceptibility to many antifungal agents and need higher dosages of amphotericin B [6, 7]. A mainstay of the successful management is the reversal of the underlying condition [4]. On the other hand, treatment of (haematologic) malignancies often leads to enhanced susceptibility for mucormycosis necessitating sophisticated management of these infections with concurrent further sufficient treatment of the malignant disease.

We describe the successful management of mucormycosis during ALL treatment.

## Case description

**Fig. 1:**
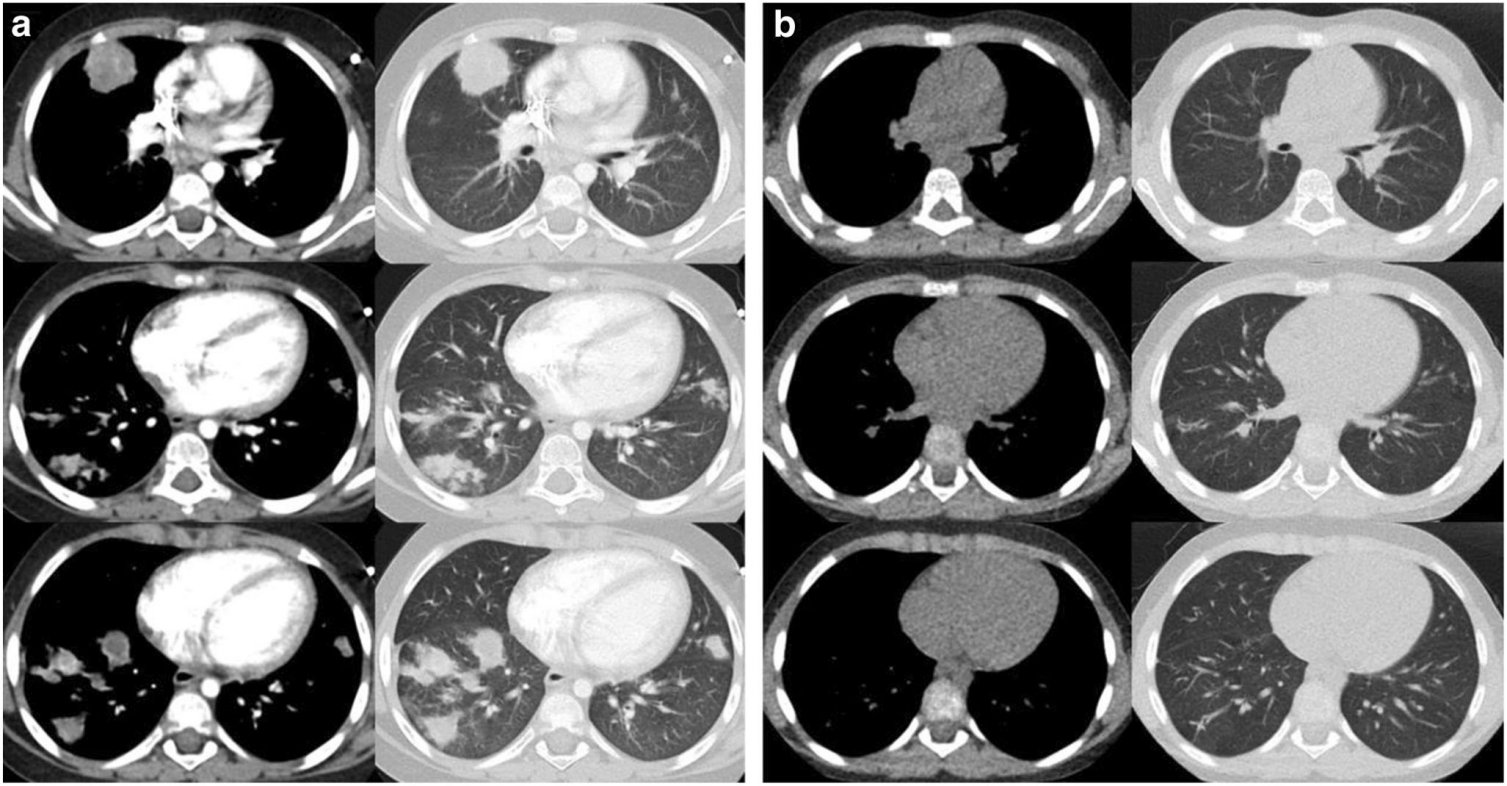
Initial (**a**) CT scan of the chest and at last follow-up (**b**). Soft tissue windowing images are displayed in the left and corresponding lung windowing images in the right rows. In the initial series (**a**), multiple, partly enhancing nodules in both lungs with a maximum diameter of 2.1 cm, predominantly in the lung periphery were detected. Furthermore, two consolidations with a maximum extent of 4.7 cm were present in the lingula and the right lower lobe. At last follow-up (**b**), only a few subtle residual changes in the lingula and the right lower lobe were seen

**Fig. 2:**
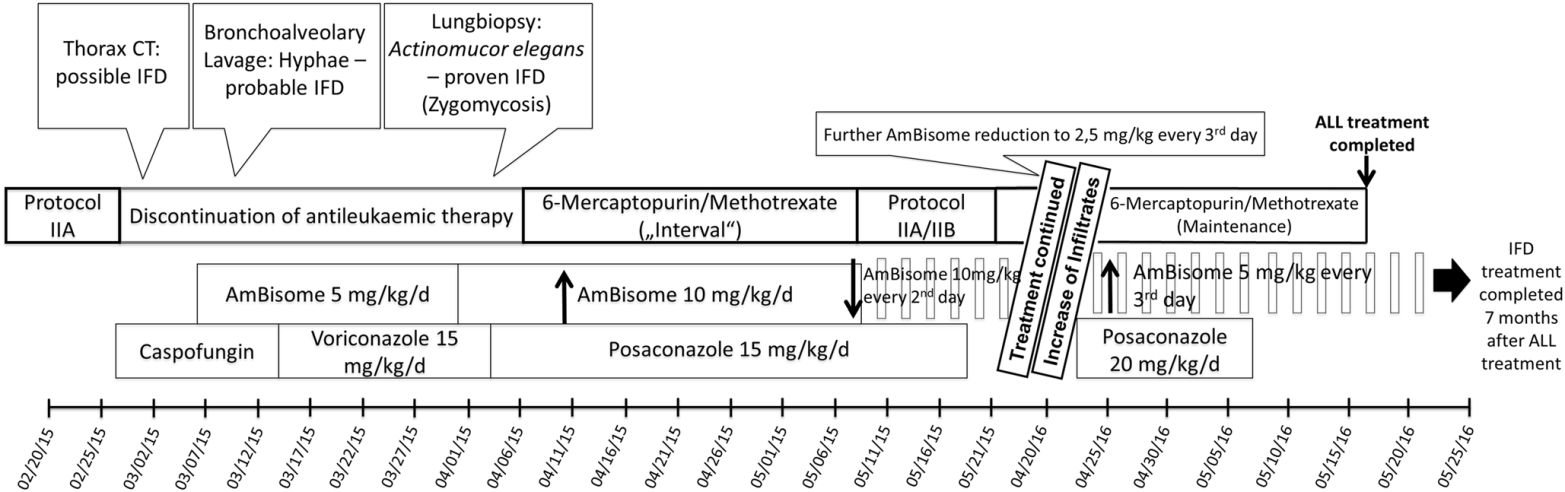
Treatment timeline

A 5-year-old patient was treated for precursor B cell acute lymphoblastic leukaemia (ALL) according to AIEOP BFM ALL 2009 protocol for 7 months in the non-high-risk arm. For digestive decolonisation, he continuously received oral amphotericin B. Overall, he had received antileukaemic treatment for 25 weeks and 3 weeks of restarted steroid therapy in protocol IIA with dexamethasone (10 mg/m^2^/day), before he developed fever and CRP levels with a maximum of 200 mg/L during neutropenia (neutrophilic leucocyte count < 500/μL); thus, ALL treatment was stopped. Chest X-ray performed 15 days after admission showed a diffuse spread infiltrate of up to 3 cm in the right middle to lower field and treatment with meropenem and clarithromycin was initiated and later switched to ceftazidim and linezolid. Furthermore, antimycotic treatment with caspofungin (1 mg/kg/d) was initiated. Bronchoalveolar lavage (BAL) was sent for microbiological evaluation including auramine staining for mycobacteria and calcofluor-white staining for fungus and revealed hyphae and weak growth of *Aspergillus fumigatus*. Neither fungal PCR from BAL fluid nor lung biopsy was performed at that time. Antifungal treatment was switched to liposomal amphotericin B (AmBisome®, 1 mg/kg/day) and voriconazole (8 mg/kg/day). Due to clinical and radiologic deterioration (Fig. 1), the patient was transferred to our University Hospital. Beta-d-glucan and galactomannan were both unremarkable, whereas CRP was still slightly elevated with 9.3 mg/L. Uncomplicated thoracoscopic biopsy and subsequent histopathological examination revealed fungal hyphae, which were identified as *Actinomucor elegans* via fungal ITS PCR and sequencing (GenBank acc. no.: gb|FJ176396.1|; Identities: 312/313 bp (99%)) representing proven invasive mucormycosis according to the EORTC criteria [8, 9]. Therefore, voriconazole was switched to posaconazole suspension (15 mg/kg/day) and dosage of AmBisome® was increased to 10 mg/kg/day, with AmBisome®-induced hypopotassaemia being substituted intravenously. Cranial magnet resonance imaging (MRI) did not show any signs of central nervous involvement of mucormycosis neither did the nasal sinuses show signs of obliteration. After 4 weeks without ALL treatment, an interval therapy with methotrexate (0.5–0.7 mg/kg once weekly) and 6-mercaptopurine (1.5 mg/kg daily) was administered for another month. Under continued antifungal treatment, we reinitiated therapy according to the AIEOP BFM ALL 2009 protocol, completed block IIB and started maintenance therapy. Five weeks after the start of maintenance therapy, AmBisome® dosages were reduced to 10 mg/kg/day every 2nd day. Another 8 weeks later, AmBisome® was reduced to 2.5 mg/kg/day every 3rd day due to practicability while posaconazole had to be discontinued due to lacking compliance (Fig. 2). The pulmonary mucormycosis was monitored every 2–6 months, either by thoracic CT or by MRI. Four months after the start of antifungal treatment, we additionally performed a PET-CT scan still showing multifocal metabolically active consolidations in both lungs. After 12 months of antifungal therapy with slight radiologic improvement, CT scan again showed an increase of infiltrates, leading to an increase in AmBisome® dosages to 5 mg/kg/day every 3rd day and reinitiation of posaconazole at an increased dosage of 20 mg/kg/day. The patient again refused posaconazole intake, so we had to stop combination therapy after only 2 weeks while continuing AmBisome® at 5 mg/kg/day every 3rd day. ALL treatment was terminated 22 months after diagnosis (2 months earlier than required) in order to improve the immune system. Flow cytometry further showed diminished B and T cell fractions after the termination of ALL treatment and CT/MRI as well as PET-CT scans still indicated infiltrates with reduced but still recognisable activity. Therefore, AmBisome® was given for another 7 months, until immunologic reconstitution (indicated by flow cytometry) and imaging showed significant improvement. Thirty months after the end of antileukaemic treatment and 23 months after the end of antifungal treatment, the patient is still in the first remission of ALL and recent thoracic CT and chest X-ray indicate further improvement with only signs of tissue scarring. An overview of our management is depicted in Fig. 2.

## Discussion

We report the successful management of a 5-year-old patient treated for ALL, complicated by an opportunistic infection with *Actinomucor elegans* representing the first paediatric patient with this pathogen.

*Actinomucor elegans* belongs to the genus *Actinomucor* in the order *Mucorales*. So far, only 5 cases of infection with *Actinomucor elegans* in humans have been described in adults [2, 10, 11]. Two patients (1 immunocompetent, 1 recipient of an allogeneic stem cell transplantation for lymphoma) were described with sinusitis and were cured with debridement, irrigation and antifungal treatment [2, 12]. Two patients were described with soft tissue infections. One of them with underlying refractory aplastic anaemia died despite debridement and antifungal treatment [11]. In the other patient, treatment and outcome were not reported [13]. Another patient developed disseminated disease after extensive wounds and died despite extensive debridement and antifungal treatment [14].

While mucormycosis in patients with diabetes mainly causes rhinocerebral and sino-orbital infections, patients with malignancy typically develop pulmonary infections. The extensive angioinvasive properties result in vessel thrombosis, tissue necrosis and haematogenous dissemination. Furthermore, ischaemic necrosis prevents leukocyte and drug penetration [4].

In our case, host factors and imaging (representing possible IFD [8, 15]) led to the initiation of antifungal treatment. The growth of *Aspergillus fumigatus* from BAL fluid retrospectively most likely represents airway colonisation, reflecting the low specificity of cultivation of moulds from BAL fluid [1]. The detection of hyphae from otherwise sterile material and molecular analyses led to the diagnosis of proven mucormycosis [9].

Currently no indirect methods are available facilitating the diagnosis [16], since cell walls of *Mucorales* spp*.* are lacking (1–3)-beta-d-glucan and galactomannan, which therefore cannot be used to diagnose invasive mucormycosis [1, 4, 17].

Initial empiric antifungal therapy comprising caspofungin, conventional doses of liposomal amphotericin B and voriconazole did not prevent deterioration. After proof of mucormycosis, therapy was switched to a combination of high-dose liposomal amphotericin B and posaconazole; despite lacking clear evidence, we decided for combination therapy due to the extent of the pulmonary lesions and the poor prognosis of invasive mucormycoses. Due to the widespread diffuse character of the infiltrates, we abandoned thoracotomy and surgical debridement. *Mucorales* spp. are considered to be resistant against echinocandins as well as against most azoles like voriconazole, fluconazole and itraconazole [7, 18] and require higher doses of amphotericin B usually administered in the liposomal formulation (AmBisome®) [6, 19]. Early treatment with high doses of amphotericin B is important since a delay results in a twofold increase in mortality leading to an overall mortality of up to 100% for patients with disseminated disease [4]. The optimal dosage has still not been determined. Many clinicians use the maximum tolerable dosages, condoning nephrotoxicity, while efficacy results from a phase II clinical trial of high-dose therapy with 10 mg/kg/day i.v. are still pending (http://clinicaltrials.gov/show/NCT00467883) [4]. Furthermore, posaconazole monotherapy is not recommended due to several reports on breakthrough mucormycoses under posaconazole prophylaxis [20].

In addition to antifungal treatment, the elimination of risk factors such as immunosuppressive therapies is essential for successful management of mucormycosis [4, 21]. As in our patient, a common risk factor is the treatment of haematologic and other malignancies, which on the other hand is vital to be cured. Therefore, it was crucial to continue treatment of ALL as soon as possible, despite invasive pulmonary mucormycosis. After initiation of adequate therapy for mucormycosis, we also reinitiated antileukaemic treatment with an interval treatment aligned to the ALL maintenance therapy and switched to the reinduction according to protocol 7 weeks later. The duration of treatment for mucormycosis remains unclear. ECIL recommends duration on an individual basis, but should continue for at least 6–8 weeks [22]. We gradually reduced dosages and prolonged intervals of AmBisome® infusions during ALL maintenance therapy to minimise side effects and to enable ambulatory parenteral therapy during several months of treatment. Intermittent AmBisome® administration is considered to be an effective alternative to the standard regimen in preventing and possibly treating invasive fungal diseases, since it appears to accumulate in tissue [23, 24]. We used this approach for a long time therapy of 1.5 years. However, after 1 year of antifungal treatment and 10 months of ALL maintenance therapy, again progression of the infiltrates was seen, which underlines the importance of radiologic monitoring and constant adequate treatment until immune reconstitution clearly has been achieved. We reintensified and successfully continued antifungal treatment with intermittent AmBisome® until numerical immune reconstitution had been indicated by flow cytometry 7 months after the end of ALL treatment. We monitored our patient via intermittent thoracic MRI in addition to CT scans to reduce radiation. To discriminate active infection from tissue scarring, we also used FDG-PET imaging, which has been described to have valuable sensitivity in detecting fungal lesions [25]. To gain immune reconstitution, one might be misled to extensively shorten maintenance therapy. However, we shortened ALL therapy by only 2 months, since it had been clearly shown that shortening therapy for ALL from 18 to 12 months leads to significantly worse outcome [26].

In conclusion, proof of mucormycetes from otherwise sterile material is essential for adequate diagnostics and therapy. Continued treatment of ALL is as important as concurrent adequate (long-term) antifungal treatment, alongside radiologic and immunologic monitoring which are essential in managing these both potentially fatal diseases.

## Abbreviations

AIEOP: Associazione Italiana Ematologia Oncologia Pediatrica
ALL: Acute lymphoblastic leukaemia
CNS: Central nervous system
CRP: C-reactive protein
CT: Computed tomography
ECIL: European Conference on Infections in Leukaemia
EORTC: European Organisation for Research and Treatment of Cancer
FDG: Fluoro-2-deoxyglucose
IFD: Invasive fungal disease
IMI: Invasive mould infection
MRI: Magnetic resonance imaging
PCR: Polymerase chain reaction
PET: Positron emission tomography
